# Synthetic antimicrobial and LPS-neutralising peptides suppress inflammatory and immune responses in skin cells and promote keratinocyte migration

**DOI:** 10.1038/srep31577

**Published:** 2016-08-11

**Authors:** Anja Pfalzgraff, Lena Heinbockel, Qi Su, Thomas Gutsmann, Klaus Brandenburg, Günther Weindl

**Affiliations:** 1 Freie Universität Berlin, Institute of Pharmacy (Pharmacology and Toxicology), Berlin, Germany; 2 Research Center Borstel, Leibniz-Center for Medicine and Biosciences, Divisions of Biophysics, Borstel, Germany

## Abstract

The stagnation in the development of new antibiotics and the concomitant high increase of resistant bacteria emphasize the urgent need for new therapeutic options. Antimicrobial peptides are promising agents for the treatment of bacterial infections and recent studies indicate that Pep19-2.5, a synthetic anti-lipopolysaccharide (LPS) peptide (SALP), efficiently neutralises pathogenicity factors of Gram-negative (LPS) and Gram-positive (lipoprotein/-peptide, LP) bacteria and protects against sepsis. Here, we investigated the potential of Pep19-2.5 and the structurally related compound Pep19-4LF for their therapeutic application in bacterial skin infections. SALPs inhibited LP-induced phosphorylation of NF-κB p65 and p38 MAPK and reduced cytokine release and gene expression in primary human keratinocytes and dermal fibroblasts. In LPS-stimulated human monocyte-derived dendritic cells and Langerhans-like cells, the peptides blocked IL-6 secretion, downregulated expression of maturation markers and inhibited dendritic cell migration. Both SALPs showed a low cytotoxicity in all investigated cell types. Furthermore, SALPs markedly promoted cell migration via EGFR transactivation and ERK1/2 phosphorylation and accelerated artificial wound closure in keratinocytes. Peptide-induced keratinocyte migration was mediated by purinergic receptors and metalloproteases. In contrast, SALPs did not affect proliferation of keratinocytes. Conclusively, our data suggest a novel therapeutic target for the treatment of patients with acute and chronic skin infections.

Owing to the alarming increase in bacterial resistance towards conventional antibiotics and the decrease in the development of new antibiotics at the same time, treatment of bacterial infections has become a major clinical problem^1^. This is particularly relevant for bacterial skin and soft-tissue infections (SSTIs) being most commonly caused by multidrug-resistant bacteria with *Staphylococcus aureus* as predominant causative Gram-positive bacterium and *Pseudomonas aeruginosa* as most frequent Gram-negative bacterium and having fatal consequences if treated unproperly^2^. Bacteria can release endotoxins like lipopolysaccharide (LPS) or other pathogenicity factors such as lipopoteins/peptides (LP) from their cell envelope, even due to treatment with conventional antibiotics, being able to activate Toll-like receptors (TLRs) and induce a strong inflammatory response. Synthetic anti-LPS peptides (SALPs) were specifically developed to neutralise these pathogenicity factors and represent an innovative approach for the treatment of bacterial sepsis^3^,^4^.

The skin comprises various cell types providing the potential to react to bacterial pathogenicity factors and can thus initiate inflammatory responses if exposed to these factors^5^. Keratinocytes are central sentinels of the skin endowed with the capability of recognising pathogen-associated molecular patterns and danger-associated molecular patterns via TLRs and the inflammasome machinery^6^. Furthermore, keratinocyte migration and proliferation are critical for re-epithelialization of skin wounds^7^. Fibroblasts residing in the dermis and thus being exposed to pathogens invading deeper layers of the skin are able to amplify cutaneous immune resonses by secreting pro-inflammatory cytokines and chemokines in cross-talk with activated keratinocytes^8^,^9^. While keratinocytes are an important part of innate immunity, antigen presenting cells (APCs) are pivotal for initiation of adaptive immune responses and regulation of T cell responses^10^,^11^. Although inflammation is an important process to combat infections and to accelerate wound healing, overactivation of the immune system can lead to detrimental effects such as chronic skin inflammation^12^.

Recent evidence has suggested that endogenous and synthetic AMPs such as LL-37 and PXL150 may be beneficial for the topical treatment of skin infections and wounds^13^,^14^,^15^. However, by acting on cell membranes their mode of antimicrobial action is rather non-specific with often poor selectivity for bacterial over mammalian cells^16^,^17^,^18^,^19^.

In this study, we investigated, if the SALP Pep19-2.5 and the structurally related compound Pep19-4LF are likewise able to reduce inflammatory and immune responses evoked by the pathogenicity factors Fibroblast-stimulating lipopeptide-1 (FSL-1) for Gram-positive bacteria and LPS for Gram-negative bacteria in different skin cells. Additionally, we tested the ability of SALPs to modulate keratinocyte migration and proliferation which are considered key events in wound healing during re-epithelialization. Our results demonstrate that synthetic anti-LPS peptides (SALPs) abrogate proinflammatory and immune responses in skin cells with the concomitant benefit of low cytotoxicity in all investigated cell types. Importantly, SALPs potently stimulate keratinocyte migration making them promising candidates for the treatment of acute and chronic bacterial skin infections.

## Results

### SALPs show low cytotoxicity in primary human keratinocytes and fibroblasts

**Figure 1:**
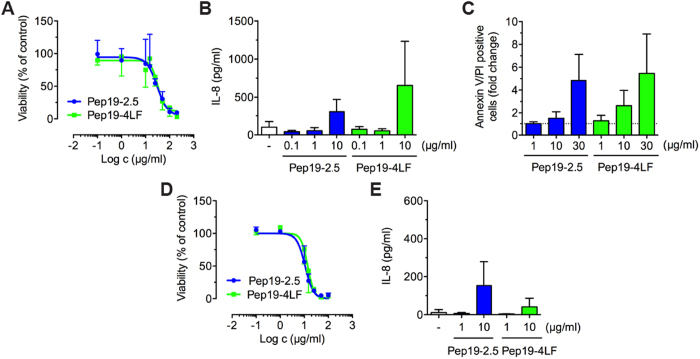
Pep19-2.5 and Pep19-4LF show low cytotoxicity in primary human keratinocytes and fibroblasts. (**A–C**,**E**) Keratinocytes and (**D**,**E**) fibroblasts were stimulated for 24 h with different concentrations of Pep19-2.5 and Pep19-4LF and analysed by cell viability assays. (**A**,**D**) Dose-response curves were obtained by MTT assay. Data are mean ± SD (n = 3-4). (**B,E**) IL-8 release into medium was quantified by ELISA. Data are mean + SD (n = 3-6). (**C**) Annexin V-FITC and PI staining followed by flow cytometry analysis. Double positive cells are displayed as fold change compared to control. Data are mean + SD (n = 3-4).

AMPs may have cytotoxic effects on human keratinocytes even at low concentrations^20^. Pep19-2.5 and Pep19-4LF showed no effects on keratinocyte growth and viability for peptide concentrations up to 10 μg/ml using the MTT assay (Fig. 1A). The IC_50_ values were 30 and 33 μg/ml for Pep19-2.5 and Pep19-4LF, respectively. Additionally, peptide concentrations below 10 μg/ml did not result in an increase of IL-8 secretion by keratinocytes (Fig. 1B) and annexin V/PI double positive cells (Fig. 1C). For primary human fibroblasts the MTT assay revealed IC_50_ values of 11 and 14 μg/ml for Pep19-2.5 and Pep19-4LF, respectively (Fig. 1D). At peptide concentrations of 10 μg/ml IL-8 secretion was increased (Fig. 1E). Thus, peptide concentrations of 1 μg/ml were used for subsequent neutralisation experiments.

### SALPs inhibit TLR2-induced responses in human keratinocytes and fibroblasts

**Figure 2:**
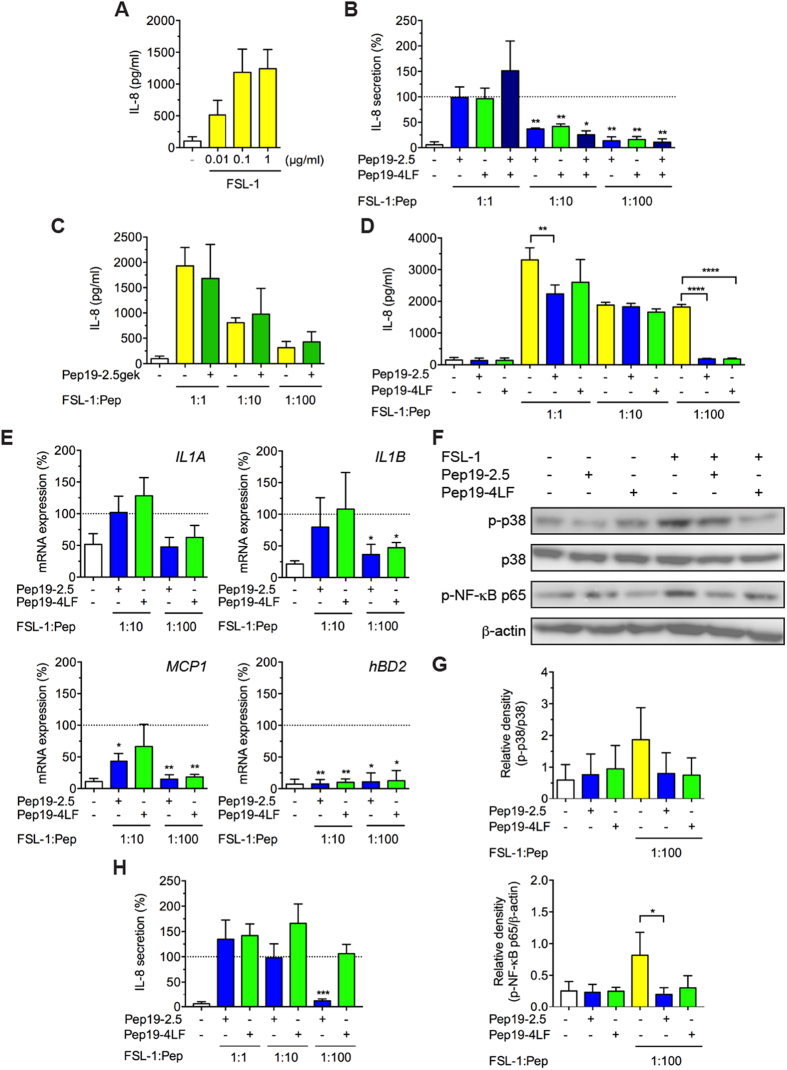
SALPs protect against FSL-1-induced responses in keratinocytes and fibroblasts. (**A**–**C**) Keratinocytes, (**D**) HaCaT cells and (**H**) fibroblasts were stimulated with FSL-1 in the presence or absence of the peptides and additionally (**D**) only peptides in the absence of FSL-1. After 24 h, supernatants were collected and IL-8 secretion was quantified by ELISA. Data are mean + SD. (**A**) (n = 6-8). (**B**,**C**,**H**) (n = 3) *p ≤ 0.05, **p ≤ 0.01, ****p ≤ 0.001, one-sample t test. (**D**) (n = 3). **p ≤ 0.01, ****p ≤ 0.001, one-way ANOVA followed by Bonferroni posttest in comparison with FSL-1-treated cells in the absence of the peptides. (**E**) Primary keratinocytes were stimulated with 0.01 or 0.1 μg/ml FSL-1 in the presence or absence of 1 μg/ml Pep19-2.5 or Pep19-4LF for 4 h and gene expression of *IL1A*, *IL1B*, *MCP1* and *hBD2* was determined by qPCR. mRNA expression values were normalised to *YWHAZ*. Data are mean + SD (n = 3). *p ≤ 0.05, **p ≤ 0.01, one-sample t test. (**B**,**E**,**H**) Data were normalised to FSL-1-treated cells in the absence of the peptides (100%). (**F**) Keratinocytes were stimulated with FSL-1 (0. 01 μg/ml) in the presence or absence of the peptides (1 μg/ml) or with peptides alone (1 μg/ml) for 30 min and phospho-p38 MAPK and phospho-NF-κB p65 were detected by Western blot analysis. Pictures are representative of three independent experiments. (**G**) Bar graphs obtained by densitometric analysis of western blot data. Mean + SD (n = 3). *p ≤ 0.05, one-way ANOVA followed by Bonferroni posttest.

Next, we investigated the capacity of the peptides to reduce TLR-induced cytokine production in primary human keratinocytes and in the human immortalised keratinocyte cell line HaCaT. In accordance with previous data indicating that keratinocytes and HaCaT cells failed to induce cytokines in response to TLR4 stimulation^21^, the TLR4 agonist LPS was not capable of provoking a significant increase of IL-8 secretion, not even in the presence of IFN-γ (data not shown). In contrast, primary human keratinocytes (Fig. 2A) and HaCaT cells (data not shown) responded to the TLR2/6 agonist FSL-1 by increased IL-8 production. IL-6 protein levels were almost not detectable after stimulation with FSL-1 in primary human keratinocytes (data not shown).

Both peptides significantly reduced FSL-1-induced IL-8 production in primary human keratinocytes at a peptide:FSL-1-ratio of 10:1 and IL-8 secretion was nearly completely abolished at a ratio of 100:1 (Fig. 2B). We also investigated the combined application of Pep19-2.5 and Pep19-4LF revealing no synergistic effect. The same experiments performed with a control peptide (Pep19-2.5gek) did not show any decrease of IL-8 secretion compared to FSL-1 alone (Fig. 2C).

In HaCaT cells, the peptides alone did not increase IL-8 levels compared to control (Fig. 2D). We observed a significant reduction of FSL-1-induced IL-8 secretion when Pep19-2.5 was added at a ratio of 1:1 and for both peptides at a Pep:FSL-1-ratio of 100:1. For a Pep:FSL-1-ratio of 10:1 both peptides were not capable of attenuating the inflammatory effect of FSL-1.

AMPs are known to activate the inflammasome and there is increasing evidence that the inflammasome is likewise involved in IL-1α secretion^22^. However, IL-1α secretion was hardly detectable in FSL-1 stimulated cells and not regulated by the peptides (data not shown). In contrast, *IL-1A* and *IL-1B* mRNA levels were upregulated after 4 h stimulation with FSL-1 while expression of both genes was downregulated in the presence of the peptides for a Pep:FSL-1 ratio of 100:1 being significant for both peptides for *IL-1B* but not *IL-1A* (Fig. 2E). Similarly, IL-18 secretion is activated via the inflammasome machinery, however, we did not observe upregulated gene levels with FSL-1 in the presence or absence of the peptides (data not shown). Furthermore, both peptides reduced gene expression of monocyte chemoattractant protein 1 (*MCP1*) significantly at a Pep:FSL-1-ratio of 100:1 whereas for Pep19-2.5 a Pep:FSL-1-ratio of 10:1 was already sufficient to decrease *MCP1* gene expression significantly (Fig. 2E). We also examined the gene regulation of the antimicrobial peptides *hBD2* and *hBD3*. Notably, for a significant downregulation of the *hBD2* gene for both peptides already a Pep:FSL-1-ratio of 10:1 was sufficient (Fig. 2E) whereas *hBD3* gene expression was not significantly increased after stimulation with FSL-1 (data not shown).

As proinflammatory cytokines are regulated through NF-κB and MAPK pathways and TLR2 signalling results in activation of the transcription factors^23^ we further investigated the phosphorylation of NF-κB p65 and p38 MAPK with Western Blot. In fact, stimulation with FSL-1 induced phosphorylation of NF-κB p65 and p38 MAPK whereas addition of the peptides markedly suppressed their phosphorylation (Fig. 2F) being significant for Phospho-NF-κB p65 after addition of Pep19-2.5 (Fig. 2G). The peptides alone had little effect on p65 and p38 phosphorylation.

Skin infections can also expand to deeper layers of the skin. Fibroblasts as major cells of the dermal layer can participate in inflammatory processes and react to TLR2 but not TLR4 agonists^9^. However, none of the peptides was able to reduce FSL-1-induced IL-8 secretion culturing the fibroblasts in DMEM as basal medium (data not shown). When the cells were cultured in RPMI Pep19-2.5 reduced FSL-1-induced IL-8 secretion significantly for a Pep:FSL-1-ratio of 100:1 while Pep19-4LF remained inactive (Fig. 2H).

### SALPs do not affect cell viability of cutanous dendritic cells and inhibit LPS-induced cytokine release

**Figure 3:**
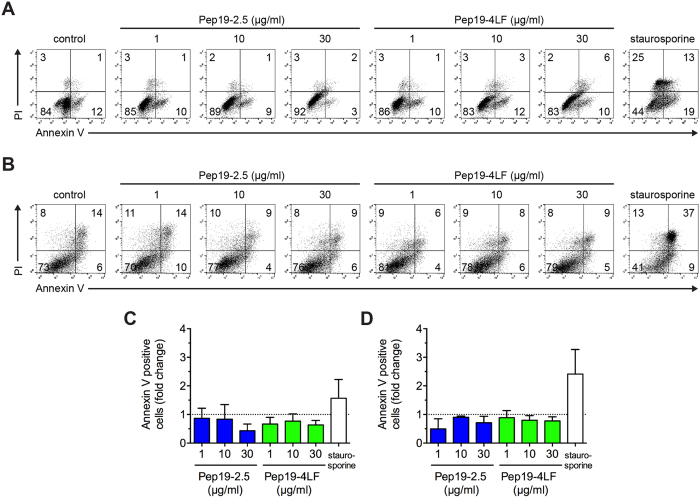
No cytotoxicity observed in antigen-presenting cells for peptide concentrations as high as 30 μg/ml. (**A**,**C**) MoDCs and (**B**,**D**) MoLCs were stimulated with Pep19-2.5 and Pep19-4LF for 24 h. Annexin V-FITC and PI staining was performed followed by flow cytometry analysis. (**A**,**B**) Dot Plots from the same donor are representative for 3 independent experiments and percentage of stained cells is indicated. Staurosporine (1 μM) served as positive control. (**C**,**D**) Annexin V-positive cells are displayed as fold change compared to control. Data are mean + SD (n = 3).

**Figure 4:**
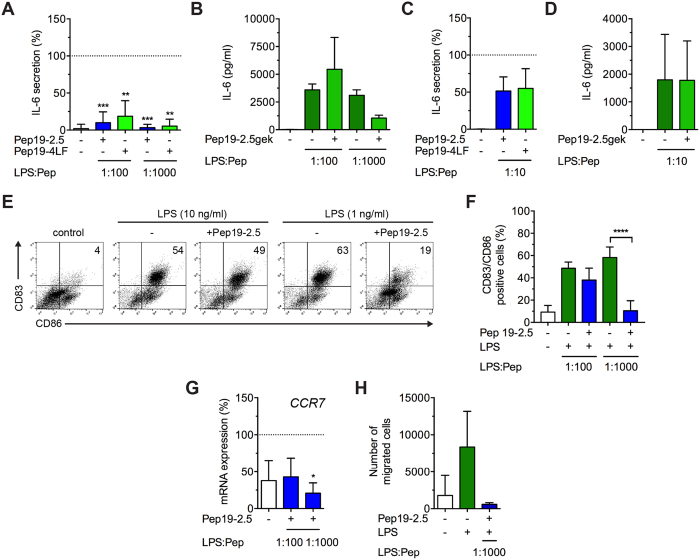
SALPs inhibit LPS-induced cytokine secretion, maturation and CCR7-dependent migration of dendritic cells. (**A**,**B**) MoDCs were stimulated with 1 or 10 ng/ml LPS (MoDCs) and (**C**,**D**) MoLCs with 100 ng/ml LPS together with 20 ng/ml TNF and 10 ng/ml IL-1β in the presence or absence of the peptides (1 μg/ml) for 24 h, supernatants were collected and IL-6 production was quantified by ELISA. Data are mean + SD (n = 3-8). *p ≤ 0.05, **p ≤ 0.01, ***p ≤ 0.001, one-sample t test. (**E**) MoDCs were stimulated with 1 or 10 ng/ml LPS in the presence or absence of Pep19-2.5 (1 μg/ml) for 24 h and surface expression of CD83 and CD86 was analysed by two-colour flow cytometry. Dot Plots are representative for 3 independent experiments and percentage of stained cells is indicated. (**F**) Bar chart summarizes means of double-positive cells. Mean + SD (n = 3). ***p ≤ 0.001, one-sample t test. (**G**) MoDCs were stimulated with 10 ng/ml LPS in the presence or absence of Pep19-2.5 for 24 h and gene expression of *CCR7* was analysed by qPCR. mRNA expression values were normalised to *YWHAZ*. Data are mean + SD (n = 3). *p ≤ 0.05, one-sample t test. (**A**,**C**,**G**) Data were normalised to LPS-treated cells in the absence of the peptides (100%). (**H**) MoDCs were stimulated with 1 ng/ml LPS in the presence or absence of Pep19-2.5 for 48 h. Cell migration was evaluated after 2.5 h towards the ligand CCL21 (100 ng/ml) by flow cytometry. Data are mean + SD (n = 4).

To determine if SALPs also neutralise LPS-induced responses in skin cells, we investigated monocyte-derived dendritic cells (MoDCs) and monocyte-derived Langerhans-like cells (MoLCs). Both peptides showed no apoptotic or necrotic effect for concentrations up to 30 μg/ml or even a lower amount of apoptotic/necrotic cells compared to the untreated control (Fig. 3A–D). For MoDCs, LPS concentrations as low as 1 ng/ml resulted in a high increase of the pro-inflammatory cytokine IL-6, while IL12p70-levels were solely upregulated in four of six donors (data not shown). Notably, both peptides inhibited LPS dependent IL-6 production significantly when added at a Pep:LPS-ratio of 100:1 and almost completely at a Pep:LPS-ratio of 1000:1 (Fig. 4A). Pep19-2.5gek showed a slight reduction for a Pep:LPS-ratio of 1000:1 (Fig. 4B) likely due to rather unspecific binding to LPS (unpublished results). Also IL12p70 was strongly decreased in donors showing LPS-induced upregulation of this cytokine (data not shown).

MoLCs respond weakly to TLR2 activation and are only responsive toward LPS under inflammatory conditions^24^. Thus, we stimulated MoLCs additionally to LPS with the proinflammatory cytokines TNF and IL-1β. Addition of the peptides resulted in a decrease of IL-6 levels at a Pep:LPS-ratio of 10:1 (Fig. 4C) although this difference failed to be significant presumably owing to the supplementary stimulation with proinflammatory cytokines^24^. No decrease of IL-6 levels was observed after addition of the control peptide Pep19-2.5gek (Fig. 4D).

### LPS-induced maturation and CCR7-dependent migration of dendritic cells is reduced by SALPs

To potently activate T cells, DCs undergo a maturation process resulting in upregulation of maturation markers and costimulatory molecules^25^. Stimulation of MoDCs with 1 and 10 ng/ml LPS resulted in almost the same amount of CD83 and CD86 double positive cells and for a Pep19-2.5:LPS-ratio of 1000:1 a significant downregulation of the maturation markers could be achieved (Fig. 4E,F). The same applies to Pep19-4LF (data not shown). In MoLCs, the LPS-induced upregulation of the maturation markers was slightly reduced by Pep19-2.5 and Pep19-4LF for a Pep:LPS-ratio of 10:1 (data not shown).

Since activation of DCs does not necessarily correlate with their migratory capability^26^, we determined the expression of the chemokine CCR7 enabling them to migrate from the periphery to secondary lymphoid organs^10^. In MoDCs, LPS-induced *CCR7* upregulation was decreased in the presence of Pep19-2.5 at a Pep:LPS-ratio of 100:1 and significantly at a Pep:LPS-ratio of 1000:1 (Fig. 4G). Additionally, we evaluated the migratory capacity of LPS-stimulated MoDCs towards the chemoattractant CCL21. Stimulation with LPS resulted in a high migratory capacity of MoDCs along the CCL21 gradient which was completely blocked by Pep19-2.5 (Fig. 4H).

### SALPs potently stimulate migration but not proliferation of human keratinocytes

**Figure 5:**
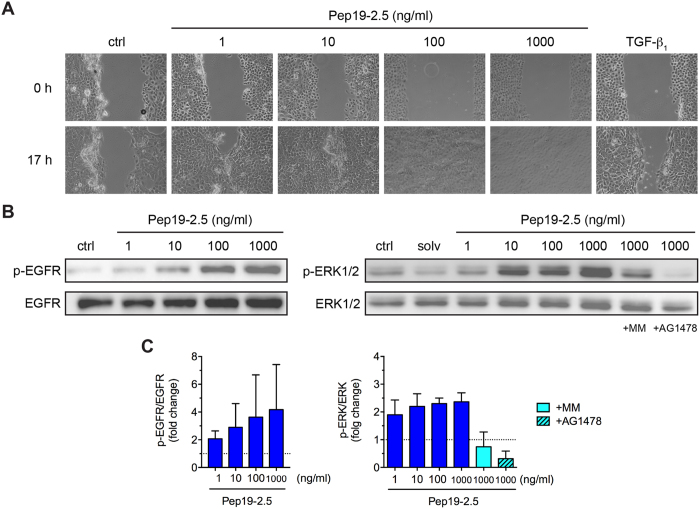
Pep19-2.5 promotes migration of primary human keratinocytes and metalloprotease/EGFR-dependent ERK1/2 activation. (**A**) Keratinocytes were scratched and stimulated with Pep19-2.5. TGF-β_1_ (1 ng/ml) served as positive control. Images were taken directly after scratching (0 h) and after 17 h and are representative of three independent experiments. (**B**,**C**) Keratinocytes were pretreated with marimastat (10 μM) or AG1478 (50 nM) and subsequently stimulated with Pep19-2.5 for 15 min. DMSO (0.1%, v/v) was used as solvent control. Expression of phospho-EGFR and phospho-ERK1/2 was detected by Western blot analysis. (**B**) Blots are representative of three independent experiments. (**C**) Bar graphs obtained by densitometric analysis of western blot data. Data are normalised to control (assigned as 1.0). Mean + SD (n = 3).

In addition to acute inflammation, bacterial infections often result in impaired wound healing^27^. Since reepithelialization is a critical step in wound repair^7^, we tested the ability of Pep19-2.5 to stimulate the migratory activity of primary human keratinocytes in the scratch wound assay. Remarkably, peptide concentrations as low as 1 ng/ml were able to markedly promote keratinocyte migration and accelerated artificial wound closure being even comparable to TGF-β_1_ (Fig. 5A), while higher concentrations were capable of completely closing the pseudo-“wound” with 1 μg/ml as optimal concentration. Since it is well known that EGFR transactivation contributes to keratinocyte migration^28^, we investigated the capacity of Pep19-2.5 to activate EGFR and EGFR-dependent extracellular signal regulated kinase 1/2 (ERK1/2) signalling. Therefore, primary human keratinocytes were stimulated with different Pep19-2.5 concentrations resulting in a dose-dependent increase in EGFR- and ERK1/2-phosphorylation (Fig. 5B). The antimicrobial peptides LL-37 and melittin provoke EGFR transactivation via metalloprotease-mediated shedding of membrane-anchored EGFR ligands^29^,^30^. Thus, we analysed whether Pep19-2.5 induces EGFR-mediated phosphorylation of ERK1/2 via metalloprotease activation. In fact, Pep19-2.5-induced phosphorylation of ERK1/2 was completely abolished in the presence of the EGFR tyrosine kinase inhibitor AG1478 and the broad-spectrum metalloproteinase inhibitor marimastat (Fig. 5B,C).

**Figure 6:**
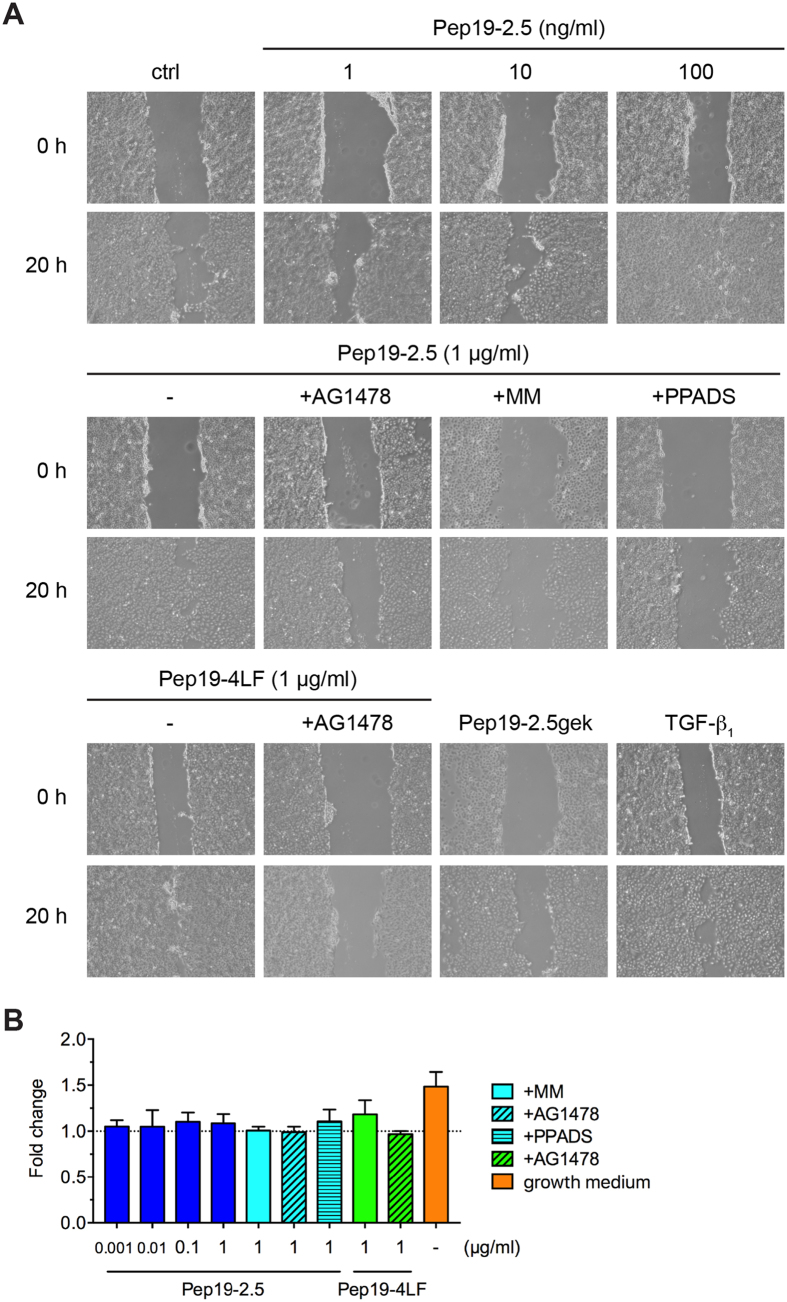
Peptide-induced keratinocyte migration depends on purinergic receptors and metalloproteases. (**A**) HaCaT cells were scratched and stimulated with Pep19-2.5 in the presence or absence of the inhibitors AG1478 (50 nM), marimastat (10 μM) and PPADS (50 μM). TGF-β_1_ served as positive control. Images were taken directly after scratching (0 h) and after 20 h and are representative of three independent experiments. (**B**) HaCaT cells were stimulated with Pep19-2.5 and Pep19-4LF in the presence or absence of the inhibitors AG1478 (50 nM), marimastat (10 μM) and PPADS (50 μM). After 24 h EdU incorporation was quantified. Data are normalised to unstimulated cells (assigned as 1.0). Cells incubated with growth medium served as positive control. Data are mean + SD (n = 3).

To confirm that the peptide-induced keratinocyte migration is due to metalloprotease-mediated EGFR transactivation, we additionally performed wound scratch assays with HaCaT cells in the presence or absence of AG1478 and marimastat. Since previous investigations with skin-derived peptides like SPINK9 revealed that purinergic receptors are involved in EGFR-transactivation^31^, we additionally used the unspecific inhibitor of purinergic receptors, PPADS. Similar to primary keratinocytes, Pep19-2.5 stimulated also HaCaT cell migration already at concentrations as low as 1 ng/ml and showed a dose-dependent increase with a maximal effect for 1 μg/ml which was blocked by AG1478, marimastat and PPADS (Fig. 6A). In HaCaT cells we further investigated the effect of Pep19-4LF and the control peptide, Pep19-2.5gek, on keratinocyte migration revealing a comparable effect for Pep19-4LF to Pep19-2.5 at a concentration of 1 μg/ml that could also be blocked by AG1478. In contrast, Pep19-2.5gek did not promote migration of HaCaT cells (Fig. 6A).

As wound closure is not exclusively due to cell migration, but also involves cell proliferation^7^, cells were pretreated with mitomycin c to exclude a potentially proliferative effect of the peptides. Furthermore, both peptides showed no or little effect on cell proliferation of HaCaT cells up to 1 μg/ml (Fig. 6B) implying that the peptide-mediated artificial wound closure is due to keratinocyte migration rather than proliferation.

## Discussion

In this study, we show that synthetic anti-LPS peptides (SALPs) abrogate TLR-induced responses in different skin cells with low cytotoxic effects. Given the importance of keratinocytes for early cutaneous innate immune responses^32^, the strong FSL-1-neutralising effect of Pep19-2.5 and Pep19-4LF provides a major contribution to prevent activation of the cutaneous immune system. SALPs might have the capability to inhibit the first inflammatory response after recognition of pathogens since they reduce the FSL-1-induced secretion of chemokines in keratinocytes. With the reduction of IL-8 they contribute to the inhibition of neutrophil recruitment to the site of infection^6^, whereas MCP-1 downregulation might attenuate monocyte infiltration^33^. This is further supported by the downregulation of the AMP hBD-2 whose secretion is upregulated in skin infections to support chemotaxis and secretion of proinflammatory cytokines^34^. As the peptides also reduce the concomitant upregulation of the proinflammatory cytokines IL-1α and IL-1β they may inhibit the subsequent activation of skin-resident immune cells that are capable to maintain and promote the ongoing immune response^6^.

The peptides showed a lower antiinflammatory effect but higher cytotoxicity in dermal fibroblasts compared to keratinocytes. The use of D-amino acid peptides revealed no further decrease of IL-8 secretion indicating that proteolytic degradation is not involved (data not shown). Although the culture medium composition affected the activity of Pep19-2.5, the underlying mechanisms remain unclear. Since we could demonstrate an involvement of Pep19-2.5 in EGFR transactivation and a role of this receptor in TLR2-mediated inflammation has been noted^35^, we assumed a possibly influence of the peptides on EGFR-induced inflammatory responses that could counteract the neutralising and thus anti-inflammatory effect in fibroblasts. However, IL-8 levels remained unchanged in fibroblasts in the presence of the EGFR tyrosine kinase inhibitor AG1478 (data not shown) suggesting no cross-talk between EGFR and TLR2 signalling pathways.

SALPs are further capable of inhibiting LPS-induced activation of dendritic cells, similarly to LL-37^36^. Since MoLCs are only responsive to TLR4 agonists under inflammatory conditions^24^ and SALPs suppress inflammatory responses in keratinocytes, LC activation might be limited in the presence of SALPs. Reduced maturation and migration of DCs results in decreased T cell activation and proliferation^36^. Consequently, detrimental local inflammatory reactions during *S. aureus* skin infections^37^ or the development of chronic inflammation dominated by a delayed lymphocyte response would seem less likely^12^.

Interestingly, the cytotoxicity of SALPs is considerably lower in MoDCs and MoLCs compared to keratinocytes and fibroblasts. Previous studies with LL-37 indicate that this AMP is able to suppress neutrophil apoptosis via activation of formyl-peptide receptor-like 1 (FPRL1) and P2X7R receptor^38^. It would be interesting to determine whether these receptors are involved in the protective effect of SALPs in cutaneous dendritic cells.

Synthetic mimics of antimicrobial peptides are able to reduce the production of proinflammatory cytokines in response to LTA, but not to other TLR2 agonists or LPS^39^. In contrast, SALPs are able to neutralise pathogenicity factors of both Gram-positive and Gram-negative bacteria giving them a broad-spectrum activity. Pep19-2.5 binds with high affinity to LPS from Gram-negative bacteria^3^,^4^ and to corresponding LP from Gram-positive bacteria or Mycoplasma^40^. Isothermal titration calorimetry revealed a strong exothermic reaction with saturation characteristics between Pep19-2.5 and LPS or LP, respectively. This interaction is connected, as has been proven for LPS^41^, with a conversion of the aggregate structure of the pathogenicity factors into a bioinactive multilamellar arrangement, which cannot be recognised by the corresponding cell surface receptors TLR2 and TLR4. A similar mode of action can be expected for Pep19-4LF. Most studies regarding antimicrobial peptides address their antibacterial effect affecting bacterial membranes and killing bacteria due to membrane-lytic processes^42^. Owing to the diversity of different bacteria it appears to be challenging to achieve a broad-spectrum activity with these AMPs. In contrast, SALPs target structures that are constituents of all bacteria species and could thus broaden the field of therapeutic application.

The strong antiinflammatory effect of SALPs - both in keratinocytes and antigen-presenting cells of the skin - makes them potential candidates for the treatment of skin and soft-tissue infections (SSTIs) or rather for the prevention of complicated SSTIs (cSSTIs). As SSTIs are the third most frequent cause for severe sepsis and septic shock^43^, making less complicated SSTIs to cSSTIs, proper treatment of these infections is indispensable. Of particular interest are infections caused by *S. aureus* becoming increasingly resistant especially towards topical antibiotics like mupirocin and fusidic acid^13^. Since AMPs target highly conserved structures of bacteria the probability of resistance development is rather unlikely^17^. Thus, SALPs could be a promising option for the treatment of infections with multiresistant bacteria, preferably in combination with antibacterial agents or other AMPs with strong antimicrobial activities, due to the low antimicrobial effect of Pep19-2.5^4^. Alternatively, a combination of Pep19-2.5 and Pep19-4LF could be considered giving a stronger antimicrobial activity of the latter SALP against Gram-negative and Gram-positive bacteria (own unpublished data), but a less inhibitory effect of the LPS-induced cytokine response in mononuclear cells^44^. In keratinocytes and dendritic cells, however, we observed a comparable pathogenicity factor neutralising effect for both SALPs. A key advantage of SALPs in this context is their ability to neutralise pathogenicity factors that can be released from bacterial envelopes after treatment with conventional antibiotics or simply due to the activation of the immune system^4^,^45^. Previous studies already show a synergistic effect of Pep19-2.5 with streptomycin for the reduction of LPS-induced TNF secretion^46^. Further studies are planned to investigate the effect of SALPs in skin infection models in combination with conventional antibiotics.

As bacterial skin infections are often associated with impaired wound healing additional wound healing properties would give a higher significance to compounds used for their treatment. Our study reveals that SALPs are effectively able to induce keratinocyte migration via EGFR transactivation, hence supporting re-epithelilization, a critical step in the wound healing process^7^. Importantly, SALPs seem far superior to LL-37 or melittin which accelerate wound closure only at much higher concentrations^28^,^30^. The Pep19-2.5-induced keratinocyte migration can be explained by the activation of metalloproteases followed by shedding of EGFR ligands and subsequent activation of EGFR which is also in accordance with previous data for the skin-derived peptide SPINK9^31^. Studies with melittin and SPINK9 point out that purinergic receptors are involved in the activation of metalloproteases^30^,^31^. Indeed, PPADS was able to decrease Pep19-2.5-induced keratinocyte migration indicating that Pep19-2.5-induced EGFR transactivation might also be due to purinergic receptor activation. Additional studies are required to characterize a possible direct interaction between purinergic receptors and SALPs.

In this study, we show that SALPs reduce the inflammatory and immune response in skin cells stimulated with potent bacterial pathogenicity factors with concurrent potent stimulation of keratinocyte migration and low cytotoxicity. Since bacterial endotoxins can give rise to prolonged increase of proinflammatory cytokines leading to chronic wound infections^47^, SALPs additionally might be a promising option for the treatment of acute and chronic wounds most commonly infected with *S. aureus* and *P. aeruginosa*^48^.

## Methods

### Cell culture

All donor and patient samples were obtained after written informed consent and only anonymised samples were used for the experiments. All experiments were performed in accordance with relevant guidelines and regulations and were approved by the ethics committee of the Charité - Universitätsmedizin Berlin, Germany. For primary cultures, normal human epidermal keratinocytes and dermal fibroblasts were isolated from human juvenile foreskin and cultured as described with minor modifications^49^,^50^. Keratinocytes were grown in keratinocyte basal medium (KBM; Lonza, Basel, Switzerland) supplemented with insulin, hydrocortisone, human epidermal growth factor and bovine pituitary extract (keratinocyte growth medium; KGM) as provided by the manufacturer. Fibroblasts and the immortalised keratinocyte cell line HaCaT (CLS Cell Lines Service, Eppelheim, Germany) were cultured in RPMI-1640 (Sigma-Aldrich, Taufkirchen, Germany) containing 2 mM l-glutamine, 100 U/ml penicillin, 100 μg/ml streptomycin (all from PAA Laboratories, Pasching, Austria) and 10% (v/v) fetal calf serum (Biochrom, Berlin, Germany). MoLCs and MoDCs were differentiated from human monocytes as previously described^24^,^51^,^52^.

### Peptide synthesis

Pep19-2.5gek and Pep19-4LF were synthesised at the Borstel Research Institute with an amidated C terminus by the solid-phase peptide synthesis technique in an automatic peptide synthesizer (model 433A; Applied Biosystems) on Fmoc-Rink amide resin, according to the 0.1-mmol FastMoc synthesis protocol of the manufacturer, including the removal of the N-terminal Fmoc group. Further details are described elsewhere^3^. Peptide 19-2.5, also termed Aspidasept^®^, was purchased from Bachem (Bubendorf, Switzerland) as research grade compound. The purity of all peptides was better than 95% as determined by HPLC and mass spectrometry. The sequences are as follows: Pep19-2.5, GCKKYRRFRWKFKGKFWFWG; Pep19-2,5gek, a shortened variant of Pep19-2.5, GCKKYRRFRWKFKGK; Pep19-4LF: GKKYRRFRWKFKGKLFLFG. The sequences of the peptides are protected in an international patent, which was granted by the European Patent Office (patent 2108 372 A1) in 2015 for EU, USA, and Japan.

### Cell stimulation

Primary cells from the 3rd passage were used and pooled from at least three donors to reduce donor-specific properties. Before stimulation, fibroblasts and HaCaT cells were washed with phosphate-buffered saline (PBS; Sigma-Aldrich) and basal medium without FCS and antibiotics was added for 24 h. In the migration studies with primary keratinocytes, KGM was changed to KBM to exclude effects of added growth factors in the cell culture medium.

Fibroblast-stimulating lipopeptide-1 (FSL-1, EMC Microcollections, Tübingen, Germany) and lipopolysaccharide (LPS) from *Salmonella enterica* Minnesota R60^4^ with or without 20 ng/ml IFN-γ (Peprotech, Hamburg, Germany) were used as TLR2 and TLR4 agonists, respectively. The peptides were preincubated with the respective stimuli for 30 min before addition to the cells. MoLCs were stimulated with LPS in the presence of 20 ng/ml TNF (eBioscience, San Diego, USA) and 10 ng/ml IL-1β (BioLegend, San Diego, USA).

### Cell viability and proliferation

Cytotoxicity was determined by the MTT assay in keratinocytes and fibroblasts^20^. Viability of untreated cells was set at 100%. IC_50_ values were calculated using GraphPad Prism 6.0 (San Diego, USA). In addition, cell death was measured by annexin V-FITC (BioLegend, San Diego, USA) and propidium iodide (PI) (Sigma-Aldrich) double staining. Cells were examined using the Cytoflex flow cytometer (Beckman Coulter, Krefeld, Germany) collecting a total of 1-2 × 10^4^ events. PI (1 μg/ml) was added to the samples directly before analysis. Staurosporine (1 μM; Tocris, Bristol, UK) served as positive control.

Proliferation of HaCaT cells was determined with EdU HTS Kit 488 (Sigma-Aldrich) according to the manufacturer’s instructions. Briefly, after 24 h stimulation cells were incubated with 10 μM 5-ethynyl-2′-deoxyuridine (EdU), fixed and permeabilised and after addition of a fluorescent dye (6-FAM Azide) EdU incorporation was determined by quantification of fluorescence intensity (FLUOstar Optima, BMG Labtech, Offenburg, Germany) (excitation: 485 nm; emission: 520 nm). All experiments were performed in triplicate.

### Flow cytometry

The cell surface expression of CD83 and CD86 was analysed in MoDCs and MoLCs by two-colour flow cytometry as described^24^.

### *In vitro* scratch assay

The scratch assay was performed as described before with minor modifications^9^. Briefly, keratinocytes and HaCaT cells were seeded in 6-well plates (TPP) and grown until they reached confluence. Cells were pretreated for 2 h with 5 μg/ml mitomycin C (Tocris) to prevent cell proliferation. After this a cell-free area was introduced by scraping the monolayer with a sterile 200-μl pipette tip. Cells were washed twice with PBS and the medium was changed to basal medium. The peptides were added in the absence or presence of the broad-spectrum metalloprotease inhibitor marimastat (MM; 10 μM; Sigma-Aldrich), the epidermal growth factor tyrosine kinase inhibitor AG1478 (50 nM; Tocris) or the non-selective P2 purinergic antagonist Pyridoxalphosphate-6-azophenyl-2′,4′-disulfonic acid tetrasodium salt (PPADS; 50 μM; Sigma-Aldrich). TGF-β_1_ (1 ng/ml, Miltenyi Biotech) served as positive control. Scratches were documented under a microscope with 10x (keratinocytes) or 5x (HaCaT cells) magnification (Axiovert 135; Carl Zeiss, Jena, Germany) equipped with a digital SLR camera (Canon EOS 1000D; Canon Germany, Krefeld, Germany) immediately after the scratching procedure and once more when kept at 37 °C and 5% CO_2_ for 17 h for primary keratinocytes and 20 h for HaCaT cells. Pictures were taken exactly at the same position before and after the incubation to document the repair process. The experiments were repeated two times and representative pictures are shown.

### Western blotting

After preincubation for 1 h with AG1478 (50 nM; Sigma-Aldrich) or MM (10 μM; Tocris) keratinocytes were stimulated for 15 or 30 min for detection of Phospho-EGFR and Phospho-ERK1/2 or Phospho-NF-κB p65 and Phospho-p38 MAPK, respectively. Subsequently, cells were lysed and prepared as described previously^9^,^52^. After gel electrophoresis and blotting membranes were blocked with 5% bovine serum albumin (BSA; Sigma-Aldrich) for 1 h at 37 °C, membranes were incubated with anti-Phospho-NF-κB p65 (Ser 536) Antibody (1:1000), anti-Phospho-p38 MAPK (Thr180/Tyr182) (D3F9) XP Rabbit mAb (1:1000), anti-Phospho-EGFR (Tyr1068) (D7A5) XP Rabbit mAb (1:1000) or anti-Phospho-p44/42 MAPK (Erk1/2) (Thr202/Tyr204) (D13.14.4E) XP Rabbit mAb (1:1000) (all from NEB, Germany) over night at 4 °C and incubated with anti-rabbit horseradish-peroxidase (HRP)-conjugated secondary antibody (NEB; 1:1000) for 1 h. Then blots were developed with SignalFire ECL reagent (NEB) and visualised by PXi Touch gel imaging system (Syngene, UK). The membranes were stripped with Restore Western Blot Stripping Buffer (Thermo Scientific) and further reprobed with anti-β-actin rabbit antibody (clone 13E5, 1:1000, NEB), anti-p38 MAPK (D13E1) XP Rabbit mAb, anti-EGFR (D38B1) XP Rabbit mAb or anti-p44/42 MAPK (Erk1/2) (137F5) Rabbit mAb (all 1:1000; NEB) to confirm comparable protein loading. Values of protein expression were measured by densitometry and normalised to β-actin-, p38 MAPK-, EGFR- or p44/42 MAPK-levels using ImageJ version 1.46r.

### ELISA

After 24 h of stimulation, cell culture media were assayed for IL-8, IL-6, IL-1α or IL-12p70 by using commercially available ELISA kits (ELISA-Ready Set Go; eBioscience).

### RNA isolation, cDNA synthesis and quantitative RT-PCR

Total RNA isolation, cDNA synthesis and quantitative RT-PCR (qPCR) were performed as described previously^49^. The following primers (synthesised by TIB Molbiol, Germany) were used: *YWHAZ*^53^, *CCR7*, *hBD2* and *hBD3*^54^ and *IL-1α* and *IL-1β*^9^ as published previously; *MCP-1*, 5′-CATTGTGGCCAAGGAGATCTG-3′ and 5′-CTTCGGAGTTTGGGTTTGCTT-3′ and *IL-18*, 5′-TGCCAACTCTGGCTGCTAAA-3′ and 5′-TTGTTGCGAGAGGAAGCGAT-3′. Fold difference in gene expression was normalised to the housekeeping gene *YWHAZ* which showed the most constant level of expression.

### *In vitro* migration assay

To analyse CCR7-dependent migration MoDCs were harvested, washed two times in PBS and stimulated with LPS in the presence or absence of Pep19-2.5 for 48 h, as described above. After washing, 5 × 10^5^ cells were added to the upper well of a 24-well transwell plate with 5 μm pore size (BD Bioscience). Complete medium in the lower well was supplemented with rh-CCL21 (100 ng/ml; Miltenyi Biotech). MoDCs were allowed to migrate for 2.5 h. The migrated cells were harvested and counted in a FACSCalibur flow cytometer for 130 s. Cell debris was excluded by scatter gates.

### Statistical analysis

Data are depicted as means + SD. Statistical significance of differences was determined by one-sample *t* test or one-way analysis of variance (ANOVA) followed by Bonferroni post-hoc analysis and considered significant at *p *≤ 0.05. Statistical analysis was performed using GraphPad Prism 6.0 (GraphPad software, San Diego, USA).

## Additional Information

**How to cite this article**: Pfalzgraff, A. *et al*. Synthetic antimicrobial and LPS-neutralising peptides suppress inflammatory and immune responses in skin cells and promote keratinocyte migration. *Sci. Rep.* **6**, 31577; doi: 10.1038/srep31577 (2016).

## References

[b1] Schäberle T. F. & Hack I. M. Overcoming the current deadlock in antibiotic research. Trends Microbiol. 22, 165–167, 10.1016/j.tim.2013.12.007 (2014). 24698433

[b2] Dryden M. S. Complicated skin and soft tissue infection. J. Antimicrob. Chemother. 65 Suppl 3, iii35–iii44, 10.1093/jac/dkq302 (2010). 20876627

[b3] Gutsmann T. . New antiseptic peptides to protect against endotoxin-mediated shock. Antimicrob. Agents Chemother. 54, 3817–3824, 10.1128/AAC.00534-10 (2010). 20606063 PMC2934961

[b4] Heinbockel L. . Preclinical investigations reveal the broad-spectrum neutralizing activity of peptide Pep19-2.5 on bacterial pathogenicity factors. Antimicrob. Agents Chemother. 57, 1480–1487, 10.1128/AAC.02066-12 (2013). 23318793 PMC3591871

[b5] Di Meglio P., Perera G. K. & Nestle F. O. The multitasking organ: recent insights into skin immune function. Immunity 35, 857–869, 10.1016/j.immuni.2011.12.003 (2011). 22195743

[b6] Nestle F. O., Di Meglio P., Qin J. Z. & Nickoloff B. J. Skin immune sentinels in health and disease. Nat. Rev. Immunol. 9, 679–691, 10.1038/nri2622 (2009). 19763149 PMC2947825

[b7] Pastar I. . Epithelialization in Wound Healing: A Comprehensive Review. Adv Wound Care (New Rochelle) 3, 445–464, 10.1089/wound.2013.0473 (2014). 25032064 PMC4086220

[b8] Sorrell J. M. & Caplan A. I. Fibroblast heterogeneity: more than skin deep. J. Cell Sci. 117, 667–675, 10.1242/jcs.01005 (2004). 14754903

[b9] Hamidi S., Schäfer-Korting M. & Weindl G. TLR2/1 and sphingosine 1-phosphate modulate inflammation, myofibroblast differentiation and cell migration in fibroblasts. Biochim. Biophys. Acta 1841, 484–494, 10.1016/j.bbalip.2014.01.008 (2014). 24440818

[b10] Said A. & Weindl G. Regulation of Dendritic Cell Function in Inflammation. J Immunol Res 2015, 743169, 10.1155/2015/743169 (2015). 26229971 PMC4503598

[b11] Joffre O., Nolte M. A., Sporri R. & Reis e Sousa C. Inflammatory signals in dendritic cell activation and the induction of adaptive immunity. Immunol. Rev. 227, 234–247, 10.1111/j.1600-065X.2008.00718.x (2009). 19120488

[b12] Karin M., Lawrence T. & Nizet V. Innate immunity gone awry: linking microbial infections to chronic inflammation and cancer. Cell 124, 823–835, 10.1016/j.cell.2006.02.016 (2006). 16497591

[b13] Mohamed M. F. & Seleem M. N. Efficacy of short novel antimicrobial and anti-inflammatory peptides in a mouse model of methicillin-resistant Staphylococcus aureus (MRSA) skin infection. Drug Des. Devel. Ther. 8, 1979–1983, 10.2147/DDDT.S72129 (2014). PMC4207544 25378910

[b14] Myhrman E. . The novel antimicrobial peptide PXL150 in the local treatment of skin and soft tissue infections. Appl. Microbiol. Biotechnol. 97, 3085–3096, 10.1007/s00253-012-4439-8 (2013). 23053090 PMC3602619

[b15] Carretero M. . *In vitro* and *in vivo* wound healing-promoting activities of human cathelicidin LL-37. J. Invest. Dermatol. 128, 223–236, 10.1038/sj.jid.5701043 (2008). 17805349

[b16] Marr A. K., Gooderham W. J. & Hancock R. E. Antibacterial peptides for therapeutic use: obstacles and realistic outlook. Curr. Opin. Pharmacol. 6, 468–472, 10.1016/j.coph.2006.04.006 (2006). 16890021

[b17] Jenssen H., Hamill P. & Hancock R. E. Peptide antimicrobial agents. Clin. Microbiol. Rev. 19, 491–511, 10.1128/CMR.00056-05 (2006). 16847082 PMC1539102

[b18] Aoki W. & Ueda M. Characterization of Antimicrobial Peptides toward the Development of Novel Antibiotics. Pharmaceuticals (Basel) 6, 1055–1081, 10.3390/ph6081055 (2013). 24276381 PMC3817730

[b19] Seil M., Nagant C., Dehaye J.-P., Vandenbranden M. & Lensink M. F. Spotlight on Human LL-37, an Immunomodulatory Peptide with Promising Cell-Penetrating Properties. Pharmaceuticals 3, 3435–3460, 10.3390/ph3113435 (2010).

[b20] Do N. . Cationic membrane-active peptides-anticancer and antifungal activity as well as penetration into human skin. Exp. Dermatol. 23, 326–331, 10.1111/exd.12384 (2014). 24661024

[b21] Köllisch G. . Various members of the Toll-like receptor family contribute to the innate immune response of human epidermal keratinocytes. Immunology 114, 531–541, 10.1111/j.1365-2567.2005.02122.x (2005). 15804290 PMC1782101

[b22] Yazdi A. S. & Drexler S. K. Regulation of interleukin 1alpha secretion by inflammasomes. Ann. Rheum. Dis. 72 Suppl 2, ii96–ii99, 10.1136/annrheumdis-2012-202252 (2013). 23253918

[b23] Krishna S. & Miller L. S. Innate and adaptive immune responses against Staphylococcus aureus skin infections. Semin. Immunopathol. 34, 261–280, 10.1007/s00281-011-0292-6 (2012). 22057887 PMC5937532

[b24] Said A., Bock S., Müller G. & Weindl G. Inflammatory conditions distinctively alter immunological functions of Langerhans-like cells and dendritic cells *in vitro*. Immunology 144, 218–230, 10.1111/imm.12363 (2015). 25059418 PMC4298416

[b25] Hemmi H. & Akira S. TLR signalling and the function of dendritic cells. Chem. Immunol. Allergy 86, 120–135, 10.1159/000086657 (2005). 15976491

[b26] Bechetoille N., Boher A., Gaydon A. & Andre-Frei V. Modulation of CD86 expression in skin dendritic cells does not always correlate with changes in DC motility, migration and allostimulatory functions. Eur. J. Dermatol. 20, 181–185, 10.1684/ejd.2010.0881 (2010). 20153995

[b27] Misic A. M., Gardner S. E. & Grice E. A. The Wound Microbiome: Modern Approaches to Examining the Role of Microorganisms in Impaired Chronic Wound Healing. Adv Wound Care (New Rochelle) 3, 502–510, 10.1089/wound.2012.0397 (2014). 25032070 PMC4086514

[b28] Tokumaru S. . Induction of keratinocyte migration via transactivation of the epidermal growth factor receptor by the antimicrobial peptide LL-37. J. Immunol. 175, 4662–4668, 10.4049/jimmunol.175.7.4662 (2005). 16177113

[b29] Kim da J. . Efficacy of the designer antimicrobial peptide SHAP1 in wound healing and wound infection. Amino Acids 46, 2333–2343, 10.1007/s00726-014-1780-5 (2014). 24952727

[b30] Sommer A. . Melittin modulates keratinocyte function through P2 receptor-dependent ADAM activation. J. Biol. Chem. 287, 23678–23689, 10.1074/jbc.M112.362756 (2012). 22613720 PMC3390642

[b31] Sperrhacke M. . SPINK9 stimulates metalloprotease/EGFR-dependent keratinocyte migration via purinergic receptor activation. J. Invest. Dermatol. 134, 1645–1654, 10.1038/jid.2014.23 (2014). 24441102

[b32] Pasparakis M., Haase I. & Nestle F. O. Mechanisms regulating skin immunity and inflammation. Nat. Rev. Immunol. 14, 289–301, 10.1038/nri3646 (2014). 24722477

[b33] Shi C. & Pamer E. G. Monocyte recruitment during infection and inflammation. Nat. Rev. Immunol. 11, 762–774, 10.1038/nri3070 (2011). 21984070 PMC3947780

[b34] Afshar M. & Gallo R. L. Innate immune defense system of the skin. Vet. Dermatol. 24, 32–38 e38-39, 10.1111/j.1365-3164.2012.01082.x (2013). 23331677

[b35] Xu X. . Activation of epidermal growth factor receptor is required for NTHi-induced NF-kappaB-dependent inflammation. PloS one 6, e28216, 10.1371/journal.pone.0028216 (2011). 22132240 PMC3223233

[b36] Kandler K. . The anti-microbial peptide LL-37 inhibits the activation of dendritic cells by TLR ligands. Int. Immunol. 18, 1729–1736, 10.1093/intimm/dxl107 (2006). 17041145

[b37] Mölne L., Corthay A., Holmdahl R. & Tarkowski A. Role of gamma/delta T cell receptor-expressing lymphocytes in cutaneous infection caused by Staphylococcus aureus. Clin. Exp. Immunol. 132, 209–215 (2003). 12699407 10.1046/j.1365-2249.2003.02151.x PMC1808706

[b38] Nagaoka I., Tamura H. & Hirata M. An antimicrobial cathelicidin peptide, human CAP18/LL-37, suppresses neutrophil apoptosis via the activation of formyl-peptide receptor-like 1 and P2X7. J. Immunol. 176, 3044–3052, 10.4049/jimmunol.176.5.3044 (2006). 16493063

[b39] Som A. . Identification of synthetic host defense peptide mimics that exert dual antimicrobial and anti-inflammatory activities. Clin. Vaccine Immunol. 19, 1784–1791, 10.1128/CVI.00291-12 (2012). 22956655 PMC3491551

[b40] Martinez de Tejada G. . Lipoproteins/peptides are sepsis-inducing toxins from bacteria that can be neutralized by synthetic anti-endotoxin peptides. Sci. Rep. 5, 14292, 10.1038/srep14292 (2015). 26390973 PMC4585737

[b41] Kaconis Y. . Biophysical mechanisms of endotoxin neutralization by cationic amphiphilic peptides. Biophys. J. 100, 2652–2661, 10.1016/j.bpj.2011.04.041 (2011). 21641310 PMC3117184

[b42] Toke O. Antimicrobial peptides: new candidates in the fight against bacterial infections. Biopolymers 80, 717–735, 10.1002/bip.20286 (2005). 15880793

[b43] Eckmann C. & Dryden M. Treatment of complicated skin and soft-tissue infections caused by resistant bacteria: value of linezolid, tigecycline, daptomycin and vancomycin. Eur. J. Med. Res. 15, 554–563 (2010). 21163730 10.1186/2047-783X-15-12-554 PMC3352104

[b44] Brandenburg K., Heinbockel L., Correa W. & Lohner K. Peptides with dual mode of action: Killing bacteria and preventing endotoxin-induced sepsis. Biochim. Biophys. Acta 1858, 971–979, 10.1016/j.bbamem.2016.01.011 (2016). 26801369

[b45] Prins J. M., van Deventer S. J., Kuijper E. J. & Speelman P. Clinical relevance of antibiotic-induced endotoxin release. Antimicrob. Agents Chemother. 38, 1211–1218 (1994). 8092816 10.1128/aac.38.6.1211 PMC188188

[b46] Schuerholz T., Brandenburg K. & Marx G. Antimicrobial peptides and their potential application in inflammation and sepsis. Crit. Care 16, 207, 10.1186/cc11220 (2012). 22429567 PMC3681352

[b47] Ki V. & Rotstein C. Bacterial skin and soft tissue infections in adults: A review of their epidemiology, pathogenesis, diagnosis, treatment and site of care. Can. J. Infect. Dis. Med. Microbiol. 19, 173–184 (2008). 19352449 10.1155/2008/846453 PMC2605859

[b48] Serra R. . Chronic wound infections: the role of Pseudomonas aeruginosa and Staphylococcus aureus. Expert Rev. Anti Infect. Ther. 13, 605–613, 10.1586/14787210.2015.1023291 (2015). 25746414

[b49] Weindl G., Castello F. & Schäfer-Korting M. Evaluation of anti-inflammatory and atrophogenic effects of glucocorticoids on reconstructed human skin. Altern Lab Anim 39, 173–187 (2011). 21639680 10.1177/026119291103900212

[b50] Bätz F. M. . Esterase activity in excised and reconstructed human skin--biotransformation of prednicarbate and the model dye fluorescein diacetate. Eur. J. Pharm. Biopharm. 84, 374–385, 10.1016/j.ejpb.2012.11.008 (2013). 23201050

[b51] Said A., Bock S., Lajqi T., Müller G. & Weindl G. Chloroquine promotes IL-17 production by CD4+ T cells via p38-dependent IL-23 release by monocyte-derived Langerhans-like cells. J. Immunol. 193, 6135–6143, 10.4049/jimmunol.1303276 (2014). 25385822

[b52] Bock S., Pfalzgraff A. & Weindl G. Sphingosine 1-phospate differentially modulates maturation and function of human Langerhans-like cells. J. Dermatol. Sci. 82, 9–17, 10.1016/j.jdermsci.2016.01.002 (2016). 26803226

[b53] Weindl G. . Human epithelial cells establish direct antifungal defense through TLR4-mediated signaling. J. Clin. Invest. 117, 3664–3672, 10.1172/JCI28115 (2007). 17992260 PMC2066194

[b54] Bock S., Murgueitio M. S., Wolber G. & Weindl G. Acute myeloid leukaemia-derived Langerhans-like cells enhance Th1 polarization upon TLR2 engagement. Pharmacol. Res. 105, 44–53, 10.1016/j.phrs.2016.01.016 (2016). 26794428

